# The Week's Work

**Published:** 1911-09-16

**Authors:** 


### The Week's Work.

### THE LATEST RECRUIT TO THE UNIFORM SYSTEM.

A special and important meeting of the Governors of the Beckett Hospital, Barnsley, which was presided over by the Rev. T. T. Taylor, vice-president, was recently held to appoint the new committee and to make an important change in the manner of presenting the annual accounts. Although the balance sheet showed a deficit of some £224, it was explained that, owing to the death of the late collector, large subscriptions had not been got in for last year. The Governors appointed the new committee, viz.: The Rev. T. T. Taylor, Rev. W. R. Hervey (Rector of Barnsley), Mr. G. A. Bond, Mr. A. Whitham, Mr. F. Brown, Mr. J. Elliott, Mr. Ald. Rose, Mr. H. Feasby, Mr. T. E. Holdsworth, Mr. A. Smith, Mr. W. J. Jones, and Mr. G. Booth. Mr. G. A. Bond was elected chairman of committee, and Mr. A. Whitham deputy chairman. The Committee recommended the alteration of the financial year, and it was resolved that the accounts of the hospital be made up to December 31 next, and afterwards yearly up to December 31, so as to enable them to be compared with the accounts of other hospitals of the same class. This will enable the accounts to be kept on similar lines as recommended by "Burdett's Hospitals and Charities." The amount contributed this year from the Saturday and Sunday Fund was £2,100, which forms a very creditable, indeed, now, a staple, part of the hospital's income. It will be remembered that on August 12 (page 487) The Hospital published a description, "How we adopted the Uniform System of Accounts," by the Secretary of East Suffolk and Ipswich Hospital.

### HOSPITAL DISPENSERS AND AMMONIA POISONING.

The death last week of the lady who, "by misadventure," took a dose of Scrubb's ammonia in mistake for magnesia draws renewed attention to the Order in Council, noted in The Hospital on July 15 (page 390), by which, on and after February 1, 1912, "preparations containing more than five per cent. by weight of free ammonia must be sold in bottles distinguishable by touch from ordinary bottles and which must be labelled with the word 'Poisonous' and with the words 'Not to be taken,' in addition to the name and address of the seller." Our comment contained the hope that the annual death-rate from ammonia poisoning would be diminished, and hospital dispensers will probably agree that a regular intercourse with out-patients such as theirs proves how sadly in need this class of the population is of any help that the Legislature can afford to its uneducated carelessness in the matter of handling and consuming pharmaceutical preparations.

### AN ADDITION TO LEICESTER INFIRMARY.

Sir Edward Wood, in a speech directed to the Hospital Saturday Society of Leicester, called attention to one or two disadvantages from which the Leicester Infirmary was suffering, disadvantages which had been emphasised by the unusual heat of the present summer. These were curable by a system of refrigeration and cold storage, the need for which had impressed him so forcibly that he had given instructions for a system to be installed at a cost of £250. There is no doubt that one of the principal lessons of the year for hospital committee-men to grasp has been enforced not only by the heat but by the strike—namely, the great desirability of every hospital of any size being independent of external ice-supply. We think there will be a general movement to instal an ice-plant in all the larger hospitals of the country.

### A BEQUEST WITH A COMMON CONDITION.

The anxiety of people concerning the careful tending of their own graves and monumental masonry may or may not be a healthy one, but it is a foible interesting to institutional workers because it leads not infrequently to bequests to hospitals. These are expected to have longer memories and to be more careful not to lose their legacies than private people. Thus the late Mr. George Ludlow Lopes, a former army officer, who lived near Bath, has left £1,000 upon trust and to pay the income to the Bath Royal United Hospital on condition and so long as "it shall keep clean and maintain in good repair the mausoleum to be erected to my memory in Westbury churchyard." If the Royal United Hospital fails to observe this condition the bequest is to revert, apparently unconditionally, to the Devon and Cornwall Hospital.

### INFIRMARY HOSTELS FOR MEDICAL STUDENTS

What are described as the first hostels for medical students to be opened in Scotland directly connected with a hospital, have now been started at Glasgow, as an adjunct to the Western Infirmary. The initial expenses of the two hostels have been guaranteed to some extent by the gift of £1,000 from various donors, and so much have medical students appreciated the additional clinical opportunities and the collegiate comforts offered that both are full for the coming winter! The committee in charge, which consists of Sir Donald and Lady MacAlister, Professor Muir, Dr. Mackintosh, M.V.O., Mr. Gourlay, and Dr. and Mrs. Crawford Renton, has aimed merely at paying expenses, and its inclusive charge of one guinea a week so far promises to make the hostels self-supporting. Mr. J. W. Gourlay, of 124 St. Vincent Street, Glasgow, invites subscriptions for adapting a private house capable of accommodating twenty-five students. Which was first to adopt this obviously desirable plan—the London Hospital or the Western Infirmary, Glasgow?

### A NEW EAR AND THROAT HOSPITAL FOR PLYMOUTH.

In Plymouth the work of the Devon and Cornwall Ear and Throat Hospital has been for some time carried on in North Street under considerable difficulties, since the ever-increasing use of the hospital has made the present premises quite inadequate. It has now been decided to erect a modern and more spacious hospital on the same site. The most pressing need has been for further out-patient accommodation; it has not been an uncommon sight to see those seeking advice crowded out into the garden and even into the street. The Plymouth Borough Council has just sanctioned the use of Beaumont House in Beaumont Park for the purpose of carrying on the work of the hospital while the new buildings are being constructed. Beaumont House stands in a small park in a very accessible part of the town in the comparatively new residential quarter on the east side opposite the London and South-Western Railway terminus, and not far from the South Devon and East Cornwall General Hospital. On the new building it is proposed to spend about £2,000, but that will only provide for half of the scheme. At the present time the committee has at its disposal nearly all that sum accruing from various legacies, and a beginning will be made forthwith; the completion of the rebuilding scheme will be deferred until the financial support afforded to the institution warrants the further extension. Plans for the new building have been prepared by Messrs. Thornely, Rooke, and Barron, and their task has been to provide for the retention of the present upper floors, while making the lower floor a suitable basis for a thoroughly modern hospital when the time arrives for the demolition of the upper and older parts. A good deal of the present garden ground is to be built on, due regard being given to the requirements of the Corporation as to air space, and a free current of air round the building is to be assured. This has enabled the architects nearly to re-plan the ground floor, which is used almost entirely for out-patients, for whom there will be a separate entrance. The plans of the out-patients' department have been arranged on modern lines; the patients will proceed from the entrance to the exit without any retracing of steps. Although for the present there will be made only slight alterations in the upper floors, it is proposed eventually to construct two wards, each containing seven beds, together with operating room and annexes, and a third ward for out-patients who have to receive such treatment as will make desirable their detention for a short time. The draft plans have received the sanction of the Charity Commissioners, who have expressed their satisfaction with the arrangements.

### NEW COTTAGE HOSPITAL AT AXMINSTER.

The foundation stone of a new cottage hospital for Axminster was laid on September 6 by Robert Cornish, Esq., J.P., the President of the Hospital. This step is the outcome of a decision taken last November to undertake the construction of a new hospital to replace the one which had been in use for the last twenty-five years. A generous response to the appeal for funds resulted in the raising of £1,900 (including a reserve fund) towards the £2,000 which it was estimated the building would cost, and there is every prospect that when the doors of the Hospital are opened it will be free from debt. The plans have been drawn up by Mr. Leslie Moore, A.R.I.B.A., of London, while the contract for the building has been secured by Mr. R. G. Spiller, of Chard, at £1,725. The Hospital will be built of brick, covered with rough cast, with tiled roofs, and in the style of a bungalow—just a one-storey building. It will comprise an entrance hall, with Matron's sitting-room and Board-room leading off. In addition to a private ward, accident ward, and operating-room, there will be male and female wards, each containing four beds, with the usual offices. The kitchen will contain the apparatus for heating the building, and there will also be sleeping accommodation for the Matron, nurses, and servants. At the front and back will be two nice secluded gardens for the use of patients.

### A COMING HEALTH CONFERENCE.

It is announced that in June of next year there will be held in London a large and important Health Conference and Exhibition. This conference has been arranged with a view to the promotion of interest in the practical side of public health questions, especially from the standpoint of simple home life. The exhibition will be on an extensive scale, and will be patronised by influential persons and expert public workers. We understand that Miss R. Gill has undertaken the office of organising secretary and that further particulars will be announced shortly. Such a conference, efficiently organised, is capable of being turned to great practical account by institutional workers, and we hope the desirability of being represented at the meetings will strongly appeal to all classes of workers in the huge field of public hygiene. In America exhibitions of this sort are very popular, and we believe that hospital workers find it pays to support them and secure adequate representation on their committees.

### THIS WEEK'S DRUG MARKET.

Few price changes of much importance have taken place, and business in the drug market has been rather quiet. The position of opium is practically unchanged, but sellers show no inclination to lower their prices; in view of the small crop of Turkish opium, there seems little likelihood of any reduction in value for some time to come. Morphine and codeine are unchanged, and prices are firmly maintained. Apomorphine is dearer. In spite of the hot weather and consequent small demand for cod-liver oil, the prices quoted are, if anything, slightly higher. Balsam tolu is scarce and tends dearer. Extremely high prices are still quoted for hydrastis canadensis. Peppermint oil is dearer. Up to the present makers of salts of mercury have announced no reduction in their quotations, as a result of the reduction in the price of quicksilver. At the recent public sale of drugs in Mincing Lane large supplies of Jamaica honey were offered, and sold at prices only very slightly lower in spite of the large supply. Cardamoms fetched somewhat higher prices, as also did aloes and senna. The demand for citric acid continues to be large, and if the hot weather prevails much longer a further advance in price should not be unexpected. The continuance of the drought is having a very serious effect upon medicinal herb crops, and higher prices may be looked for.

